# A phenotypic screen of Marfan syndrome iPSC-derived vascular smooth muscle cells uncovers GSK3β as a new target

**DOI:** 10.1016/j.stemcr.2022.12.014

**Published:** 2023-01-19

**Authors:** Hongorzul Davaapil, Madeline McNamara, Alessandra Granata, Robyn G.C. Macrae, Mei Hirano, Martina Fitzek, J.A. Aragon-Martin, Anne Child, David M. Smith, Sanjay Sinha

**Affiliations:** 1 Department of Medicine and Wellcome-MRC Cambridge Stem Cell Institute, University of Cambridge, Cambridge CB2 0AW, UK; 2 Stroke Research Group, Department of Clinical Neurosciences, Cambridge Biomedical Campus, University of Cambridge, Cambridge CB2 0QQ, UK; 3 Experimental Medicine and Immunotherapeutics, University of Cambridge, Addenbrooke’s Hospital, Cambridge CB2 0QQ, UK; 4 Emerging Innovations, Discovery Sciences, R&D, AstraZeneca, Cambridge CB2 0AA, UK; 5 Department of Surgery and Cancer, Imperial College, Guy Scadding Building, London SW3 6LY, UK; 6 The Marfan Trust, Guy Scadding Building, London SW3 6LY, UK

**Keywords:** iPSC, disease modeling, VSMC, vascular smooth muscle cell, Marfan syndrome, MFS, drug screen, GSK3β, thoracic aneurysm

## Abstract

Marfan syndrome (MFS) is a rare connective tissue disorder caused by mutations in *FBN1*. Patients with MFS notably suffer from aortic aneurysm and dissection. Despite considerable effort, animal models have proven to be poorly predictive for therapeutic intervention in human aortic disease. Patient-derived induced pluripotent stem cells can be differentiated into vascular smooth muscle cells (VSMCs) and recapitulate major features of MFS. We have screened 1,022 small molecules in our *in vitro* model, exploiting the highly proteolytic nature of MFS VSMCs, and identified 36 effective compounds. Further analysis identified GSK3β as a recurring target in the compound screen. GSK3β inhibition/knockdown did not ameliorate the proliferation defect in MFS-VSMCs but improved MFS-VSMC proteolysis and apoptosis and partially rescued fibrillin-1 deposition. To conclude, we have identified GSK3β as a novel target for MFS, forming the foundation for future work in MFS and other aortic diseases.

## Highlights

• Developed an iPSC-based drug screen for MFS and tested 1,022 small molecules
• Identified GSK3β as a recurring target among effective small molecules
• Validated the outcome of the drug screen using six GSK3β inhibitors and siRNA
• GSK3β inhibition/knockdown decreased proteolysis and apoptosis in 4 patient lines

## Graphical abstract

**Figure:**
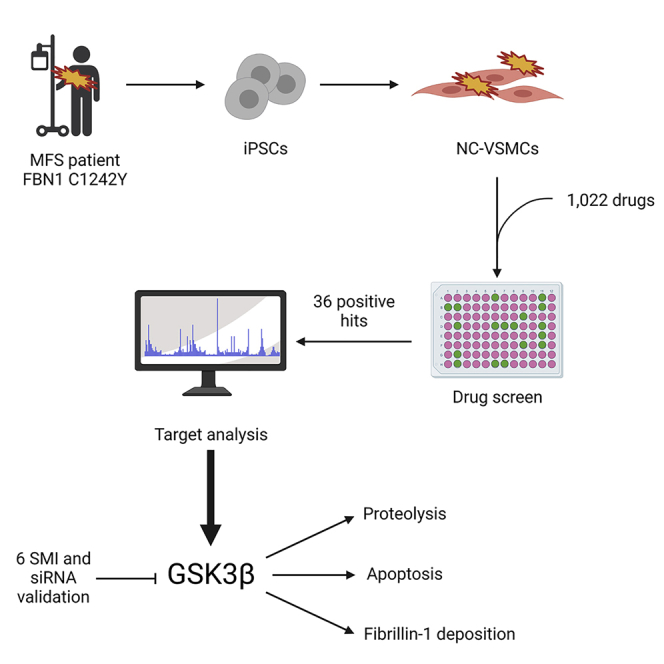

## Teaser

Animal models of MFS have not yet been successful in predicting human disease. Sinha and colleagues have developed an iPSC model of MFS and used it as a platform for drug screening. They screened over a thousand compounds and identified GSK3β as a top drug target. This work is the foundation for further screening in MFS and related diseases.

## Introduction

Marfan syndrome (MFS) is a rare genetic disorder resulting in multi-system abnormalities. It is caused by deleterious variants in the *FBN1* gene, a key extracellular matrix (ECM) protein in connective tissue (Dietz et al., 1992). The cardiovascular effects can be life threatening, as patients can develop thoracic aortic aneurysm and dissection (TAAD), particularly at the aortic root and arch. It is currently thought that the majority of aortic disease is propagated through vascular smooth muscle cells (VSMCs), although there is also evidence of endothelial dysfunction (Chung et al., 2007; Galatioto et al., 2018; Oller et al., 2017). In addition, there is heterogeneity in the embryonic origin of VSMCs present in aorta, which itself has been hypothesized to contribute to disease progression (Majesky, 2007).

The current treatment options for patients with MFS are limited to prescription of anti-hypertensives, surgical replacement, or external support (Pepper et al., 2020) of the dilated aortic root—a major procedure with significant risk of morbidity and mortality. The use of the angiotensin II receptor blocker (ARB) losartan in a mouse model of MFS was highly effective in limiting aortic disease progression (Habashi et al., 2006). Unfortunately, following up from this work, numerous clinical trials have concluded that losartan was either not successful or had only modest effects in reducing aortic diameter or improving clinical endpoints in patients (Groenink et al., 2013; Lacro et al., 2014; Milleron et al., 2015; Teixido-Tura et al., 2018). These disappointing results could be attributed to a variety of factors, including insufficient safe dosage (Mullen et al., 2020), fundamental differences between the species, and varying genetic backgrounds. There is therefore a need for alternative approaches to identify novel and effective treatment options for MFS.

Induced pluripotent stem cells (iPSCs) can be used to generate any somatic cell type, including lineage-specific VSMCs. We have developed protocols to generate lateral plate mesoderm, neural crest (NC), and paraxial mesoderm-derived VSMCs, which correspond to the aortic root, ascending aorta, and descending aorta, respectively (Cheung et al., 2012, 2014), and it is hypothesized that embryonic lineage may be important in disease susceptibility (MacFarlane et al., 2019; Majesky, 2007). Using this lineage-specific approach, an iPSC-based model of MFS *in vitro* has been developed (Granata et al., 2017). There, the main features of the aortic phenotype in VSMCs were recapitulated in VSMCs derived from the NC, notably abnormal ECM deposition, increased matrix metalloproteinase (MMP) expression and activity, apoptosis, and abnormal response to mechanical stretch. We identified p38 as a candidate for mediating the MFS phenotype, as p38 inhibition partially rescued the phenotype *in vitro* (Granata et al., 2017).

Here, we describe a medium-throughput, unbiased small molecule (SM) screen to identify novel disease mechanisms and therapeutic targets using iPSC technology. In collaboration with AstraZeneca, we have screened 1,022 SMs on MFS NC-derived VSMCs, herein referred to simply as “VSMCs,” and identified a subset that were found to reduce MMP activity. In particular, we identified that GSK3β SM inhibitors (SMIs) and genetic knockdown improved cellular function, where MFS VSMCs were less proteolytic and showed reduced apoptosis. In addition, we treated three additional MFS patient lines with a GSK3β inhibitor and obtained a consistent outcome, suggesting that this may be a common cellular defect among different MFS patient lines. This work highlights a screening strategy that could be used widely to screen additional SMIs and/or applied to models of other aortic diseases.

## Results

### Screen of 1,022 SMs

SMs are low-molecular-weight compounds typically around 500 Da in size that can modulate protein binding and activity (Khera and Rajput, 2017; Zhong et al., 2021). Because of their low molecular weight, they are able to penetrate cells more easily than macromolecular drugs, such as antibodies or other proteins. Here, we sought to screen a library of 1,022 SMs to identify any compounds that can ameliorate the disease phenotype of MFS VSMCs. The library of compounds used in this study was obtained from AstraZeneca’s Open Innovation Group. It is composed of 14,000 SMs in total and was recently used for SM screening in an iPSC model of non-alcoholic fatty liver disease (Parafati et al., 2020). These compounds are highly annotated and have information on pIC50 for primary and secondary targets; over 1,700 targets are covered in all. This library is composed of SMIs that target multiple proteins within a given signaling pathway, thereby increasing its capacity to uncover new pathways implicated in disease. As there is significant over-representation of some targets, the library of 14,000 SMIs was selectively narrowed down to 1,022 compounds in order to maintain a broad cohort of targets in a smaller number of compounds for this proof-of-concept work.

**Figure 1:**
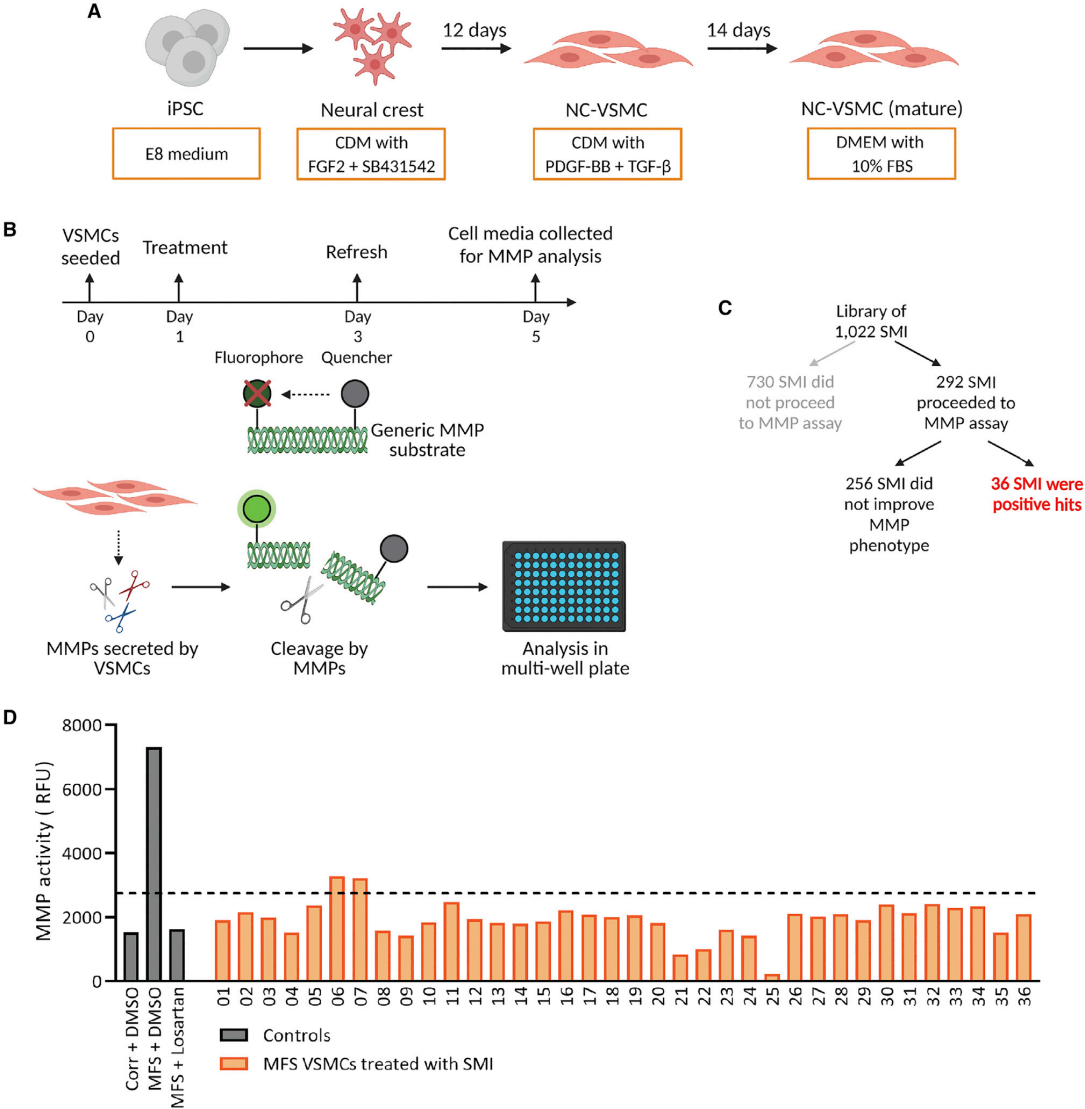
MMP activity-based drug screen (A) Overview of differentiation to NC-VSMCs from iPSCs. (B) MFS VSMCs were treated with 1 μM SMIs for 96 h and cell supernatant collected. This supernatant contains secreted MMPs, which, when incubated with the generic MMP substrate, leads to cleavage and a subsequent fluorescent signal, which can be measured using a plate reader. (C) Of the 1,022 SMIs, the majority were not suitable for further assay at 1 μM. (D) Among the SMIs used in the screen, 36 were found to decrease MMP activity of MFS VSMCs. Corrected VSMCs and MFS VSMCs treated with losartan were used as controls to determine the threshold of sufficient MMP activity reduction. Drug screen was performed as n = 2 technical replicates.

We designed this phenotypic screen around the highly proteolytic nature of MFS VSMCs, which have elevated MMP expression and secretion (Cui et al., 2021; Granata et al., 2017; Ikonomidis et al., 2006). The patient line used for this study, unless otherwise specified, is the FBN1 C1242Y line, which we have previously characterized (Granata et al., 2017). A fluorescence-quenched gelatin substrate would then be incubated with MMPs from the cell culture medium: cleavage of this substrate would result in a fluorescent signal, which can be detected in a plate reader (Figure 1B). MFS VSMCs were treated for 96 h with 1 μM SM, after which the supernatant was collected and analyzed. Of the 1,022 compounds tested at this concentration for 96 h, 730 were found to be associated with some cell atrophy and/or detachment, which made them poor candidates for proceeding to assay for MMP activity. Of the remaining 292 SMs, 36 were found to successfully reduce MMP activity down to levels comparable to the isogenic corrected control (Corr) VSMCs or MFS VSMCs treated with losartan (Figures 1C and 1D).

### GSK3β is a recurring target among the positive hits

**Figure 2:**
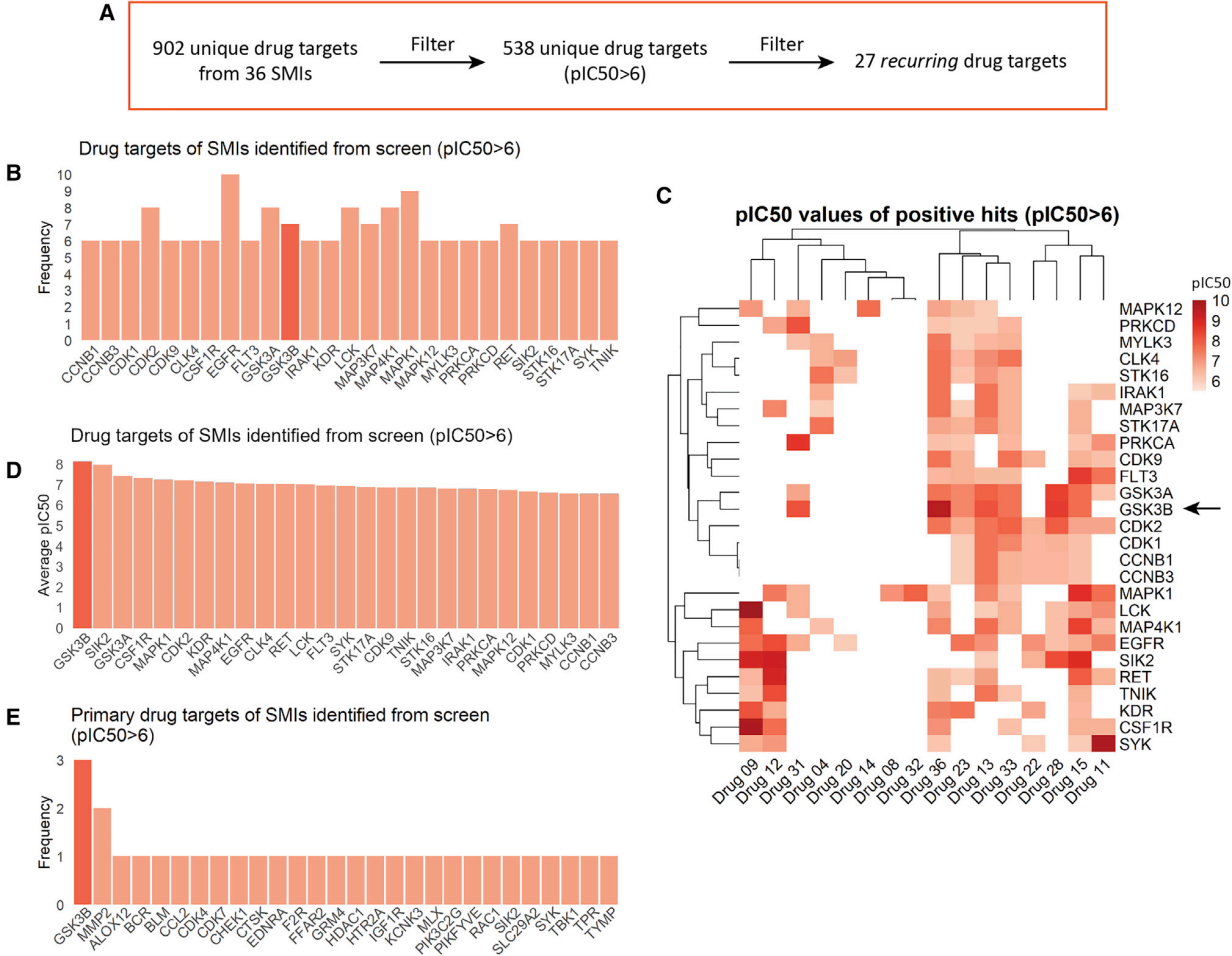
GSK3β is a recurring drug target among the positive hits from the drug screen (A) Outline of how drug targets were filtered. This was performed by removing drug targets with pIC50 <6 and then overall frequency lower than 5. (B) Frequency of drug targets suggests that GSK3β (red) is a recurring target, among others. (C) Heatmap of pIC50 values for all high-frequency drug targets indicates that GSK3β (arrow) is also a high-specificity drug target. (D) Average pIC50 values for each drug target indicates that GSK3β (red) was the most specific target. (E) Primary drug targets among the positive hits from the drug screen. GSK3β (red) is the most recurring primary target.

In order to identify a promising target worthy of further investigation, we analyzed the annotated primary and secondary targets and their associated pIC50 values (Table S1). From the 36 SMIs, we identified 902 unique targets (Figure 2A). Since SMs were used at 1 μM, pIC50 values below 6 are not informative—therefore, drug targets with a pIC50 <6 were not included in our analysis, resulting in 538 unique targets.

The majority of these targets were protein kinases, as indicated by GO term enrichment (Figure S1A). Interestingly, we identified p38 MAPK inhibitors among our positive hits along with GABA receptor inhibitors, both of which have been found to be effective in MFS by us and others (Figure S1B) (Granata et al., 2017; Hansen et al., 2019). KEGG pathway enrichment analysis indicates that components of the MAPK signal transduction pathway are highly enriched, along with other potentially interesting pathways, such as those linked to focal adhesions (Figure S1C).

Out of 538 unique targets, 27 targets were found to be present in 6 or more SMs (Figures 2B; Table S2). We identified that GSK3β is a recurrent and highly specific target, as illustrated by the heatmap (Figure 2C, arrow) and average pIC50 values (Figure 2D, red). Among the negative hits from the SM screen, i.e., compounds that did not produce a beneficial effect in MFS VSMCs, GSK3β was not a top recurring target (Figure S1D). In addition, we also found that GSK3β was the most prominent primary target among the SMIs (Figure 2E). Finally, correlation between positive and negative hits (Figure S2) also demonstrated that GSK3β is a top contender for consideration, which is emphasized by using a more stringent pIC50 threshold (Figure S2). This is particularly important as the pIC50 values from this dataset are derived from isolated enzyme activity assays—it is likely that the activity of compounds inside the cell would be lower. This reinforces our approach to use pIC50 as a cutoff but also suggests that even higher stringency may also be informative. Taken together, we therefore decided to focus our validation on GSK3β.

### GSK3β expression in Corr versus MFS cells

**Figure 3:**
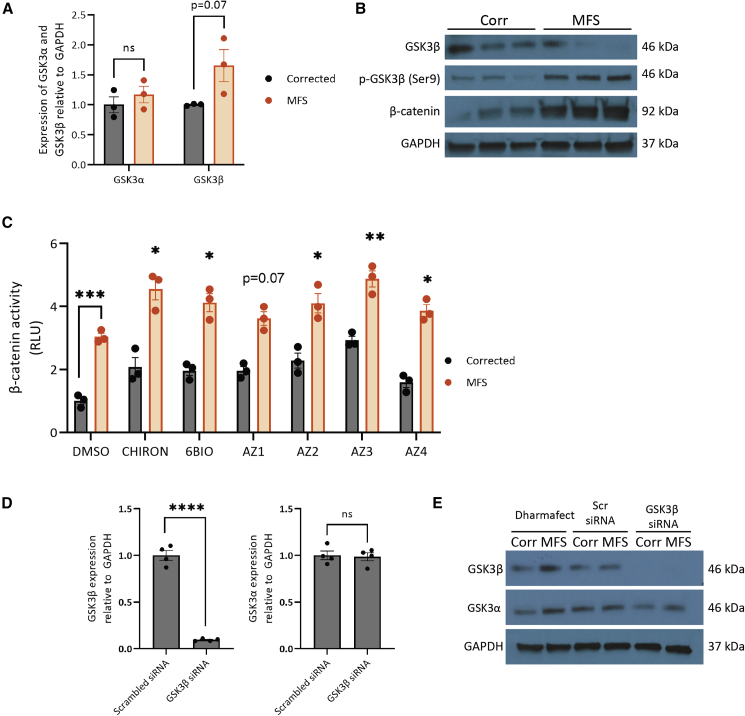
GSK3β expression in MFS VSMCs and its knockdown by siRNA (A) Expression of GSK3α and GSK3β mRNA in corrected and MFS VSMCs. (B) Expression of GSK3β, phospho-GSK3β (Ser9), and β-catenin protein in corrected and MFS VSMCs. GAPDH was used as a loading control. (C) Luciferase assay using a β-catenin reporter construct after 4 h of treatment. For the drug treatment groups, comparisons were performed with MFS DMSO. (D and E) Compared with the scrambled control siRNA, siRNA against GSK3β was effective in knocking down its expression at both the mRNA (D) and protein (E) levels without altering the expression of GSK3α. GAPDH used as the loading control. n = 3 independent experiments for qPCR, western blotting, and luciferase experiments. Corrected cells (n = 4 independent experiments) were used for siRNA qPCR analysis, and representative corrected and MFS cells were used for western blotting. Data are represented as mean ± SEM

We started by assessing the expression of GSK3β in untreated Corr and MFS VSMCs. At the mRNA level, GSK3β expression trended toward an increase (p = 0.07) in MFS cells (Figure 3A). In contrast, total GSK3β expression in MFS VSMCs is decreased compared with the Corr (Figure 3B). Interestingly, it seems that GSK3β activity is decreased, too: there is increased phosphorylation at Ser9 (Figure 3B), which is an inhibitory post-translational modification (Cross et al., 1995). This is supported by the increased amount of β-catenin in MFS cells, indicative of increased signaling through the canonical Wnt pathway. These findings were unexpected as they suggest that GSK3β activity may already be decreased in the MFS cells, yet GSK3β inhibition was identified from the drug screen as effective at reversing the MFS proteolytic phenotype.

To confirm this, we have performed a reporter assay using the M50 Super 8x TopFlash construct (Veeman et al., 2003), which contains T cell factor (TCF)/lymphoid enhancer factor (LEF) sites upstream of a luciferase reporter as a readout for β-catenin activity and hence the extent of GSK3β inhibition (Cadigan and Waterman, 2012). Even without drug treatment, we noted that there was increased β-catenin signal in MFS VSMCs compared with the corrected line, supporting our western blot findings of increased baseline inhibition of GSK3β (Figure 3C). Furthermore, after 4 h drug treatment, we also observed increased β-catenin activity, indicating that drug treatment does indeed result in further inhibition of GSK3β.

Next, in order to help with validation of GSK3β as a target, we used small interfering RNA (siRNA) to knock down the expression of GSK3β. Since SMIs frequently have multiple secondary drug targets, we used a genetic system to also verify and validate the results of our SM screen. Furthermore, knockdown was used instead of CRISPR-mediated deletion as this would not abrogate expression entirely, mimicking the effects more closely of inhibition by SMIs. siRNA-mediated knockdown of GSK3β was successful at reducing the expression of both mRNA and protein (Figures 3D and 3E). We also confirmed that this strategy did not affect the expression of GSK3α (Figure 3E).

### Decreased GSK3β reduces MMP activity and expression

**Figure 4:**
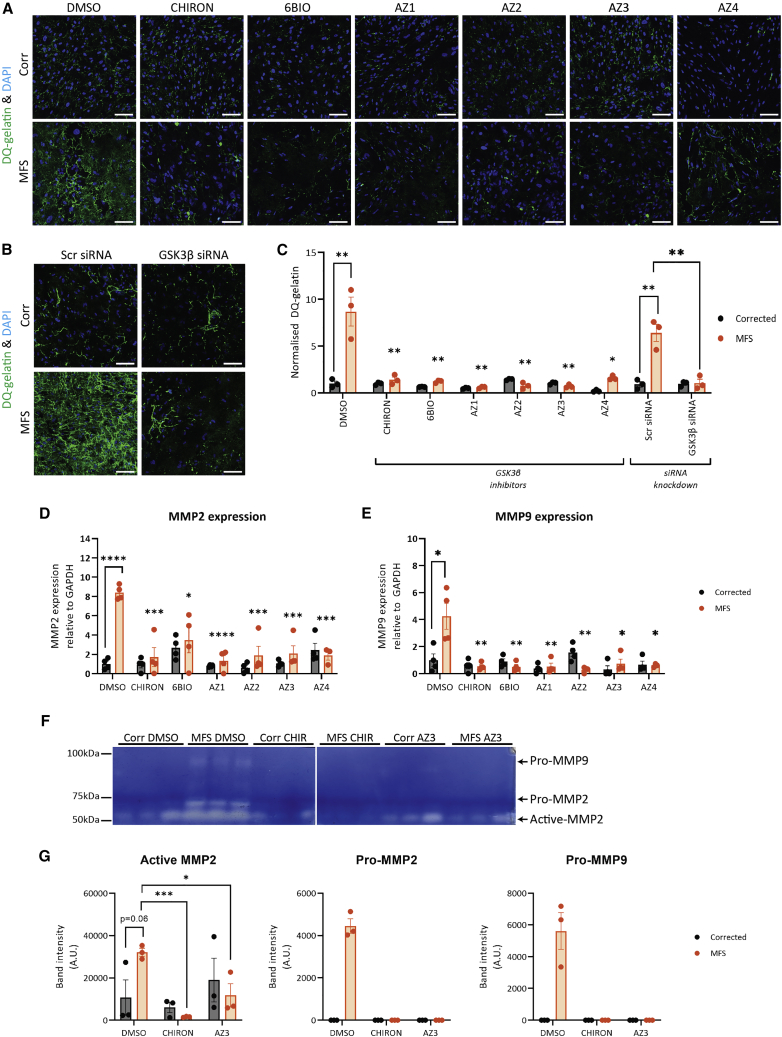
Decreased proteolysis and MMP expression upon disruption of GSK3β (A–C) *In situ* gelatin degradation assay with either treatment of GSK3β SMI 1 μM (A) or siRNA (B) and quantification (C). n = 3. (D and E) Analysis of mRNA expression of MMPs (D) 2 and (E) 9 indicate that their expression is decreased following SMI treatment; n = 3–4 independent experiments. 150 μm scale bars throughout. (F and G) Gelatin zymography following 4 days treatment with DMSO, CHIRON, and AZ3 (F) alongside band quantification (G) for active MMP2, pro-MMP2, and pro-MMP9; n = 3 independent experiments. In (C–E), comparisons were performed between MFS DMSO and drug treatment groups. Data are represented as mean ± SEM. Cells treated under control condition (DMSO) were also used as controls for Figure S3.

We then aimed to confirm the findings of the drug screen. In addition to siRNA-mediated knockdown, we decided to use six SMIs that target GSK3β: three inhibitors identified from the drug screen (6BIO, AZ1, and AZ2), along with three additional compounds (CHIRON, AZ3, and AZ4) for further validation (Table 1). We observed that upon treatment with GSK3β SMI and siRNA, there was mild initial cell death after 24 h, which did not persist thereafter. To validate the results of our screen, we performed *in situ* zymography, where we cultured cells on DQ gelatin. Similar to the MMP substrate used for the initial screen, DQ gelatin fluoresces when cleaved by MMPs, resulting in deposition of green fluorescence. After 96 h of treatment, cells were imaged, and the data were quantified in an automated and unbiased manner. Our findings indicate that while the MFS VSMCs treated with DMSO or scrambled siRNA exhibited high levels of DQ gelatin degradation, cells treated with the GSK3β inhibitors (1 μM) or siRNA showed less degradation, with levels similar to those of Corr cells (Figures 4A–4C). This successfully recapitulated the decreased matrix degradation observed when cells were treated with doxycycline, losartan, and p38 inhibitor losmapimod (Figure S3). This finding was further supported by decreased expression of MMPs 2 and 9 upon treatment with GSK3β inhibitors (Figures 4D and 4E). In our previous work, we had established that p38 inhibition is beneficial for MFS VSMCs with regards to fibrillin-1 deposition and reduced apoptosis (Granata et al., 2017)—we extend these findings to include its effects on MMP activity in our disease model.

**Table 1:** GSK3β SMI used and their pIC50 values for GSK3β

Drug	Other identifiers	pIC50	Reference
CHIRON	CHIR99021; CT 99021	9.12	Wagman et al., 2004
6BIO	6-bromoindirubin-3-oxime	8.6	Meijer et al., 2003; Polychronopoulos et al., 2004
AZ1	SN1058514991	9.5	compound from AstraZeneca
AZ2	SN1069935378	8.2	compound from AstraZeneca
AZ3	SN1030101051; AZD1080	7.9	Georgievska et al., 2013
AZ4	SN1029930290	10	compound from AstraZeneca

Furthermore, to confirm these findings, we have performed gelatin zymography. Here, cell supernatants from corrected and MFS VSMCs were harvested after 4 days of drug treatment. These supernatants were run on a gel containing gelatin to uncover the extent of gelatin degradation by secreted MMPs (Figures 4F and 4G). We noted that without drug treatment, MFS cell supernatants contained notable levels of full-length MMP9, as well as increased full-length and cleaved MMP2. Upon treatment with GSK3β inhibitors CHIRON and AZ3, there was a dramatic reduction in the MMPs in the supernatant, consistent with the findings of the DQ-gelatin assays. Taken together, these results therefore confirm that GSK3β inhibition is beneficial in decreasing the proteolytic nature of MFS VSMCs.

### GSK3β inhibition reduces apoptosis

**Figure 5:**
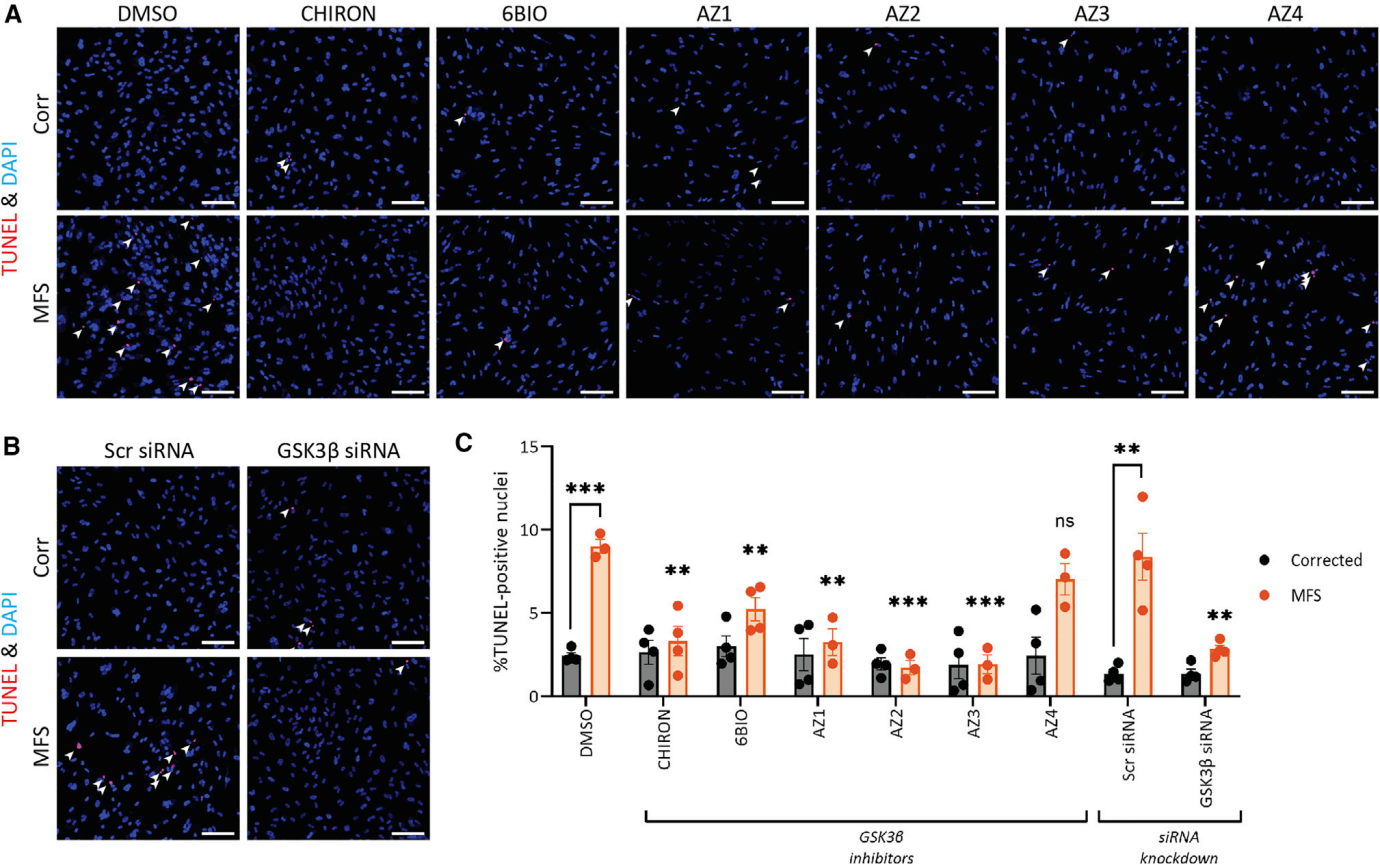
GSK3β inhibition or knockdown decreases apoptosis in MFS VSMCs (A and B) Cells were treated for 96 h prior to staining for TUNEL (red) and DAPI (blue). (C) Quantification was performed in a blinded and unbiased way using ImageJ and a macro. For the drug treatment groups, comparisons were performed with MFS DMSO. 150 μm scale bars throughout. n = 3–4 independent experiments. Data are represented as mean ± SEM. Cells treated under control condition (DMSO) were also used as controls for Figure S4.

Next, we aimed to see whether GSK3β inhibition could also decrease apoptosis using TUNEL staining. We confirmed that the assay was working by treating cells with DNase I (Figure S4A), and non-GSK3β SMIs (Figure S4B). As before, after treatment for 96 h with SMIs (1 μM) or siRNA against GSK3β, we fixed and stained cells (Figure 5). MFS VSMCs with control treatment had a higher percentage of apoptotic cells compared with the Corr. Treatment with SMIs and siRNA improved this disease phenotype, with the exception of SMI AZ4 (Figure 5). We hypothesize that the off-targets unique to AZ4 (Figure S5; Table S3) may be responsible for its diminished effectiveness in reducing the apoptotic phenotype. There are 8 unique targets: ATR, ALPG, ALPI, ALPL, ALPP, AHR, FLT1, and PRKCI. Of these targets, PRKCI has been shown to be protective against apoptosis (Flum et al., 2018; Murray and Fields, 1997; Xie et al., 2000), and therefore it is plausible that decreased activity of PRKCI in our VMSCs counteracts the beneficial effects of GSK3β inhibition. Nonetheless, the results as a whole suggest that GSK3β inhibition or knockdown is beneficial in MFS VSMCs.

### Proliferation is unaffected by GSK3β

We subsequently aimed to determine whether GSK3β inhibition could improve the proliferation phenotype by performing EdU incorporation analysis. We cultured cells in the presence of EdU for 16 h on the last day of SMI or siRNA treatment. Since the VSMCs we produce are not highly proliferative, we used HS27a cells as a positive control and confirmed that our EdU signal coincided with KI67 staining (Figure S6A). We noted that without treatment, MFS VSMCs had very poor proliferation, consistent with our experience when culturing them. In contrast, isogenic control cells had approximately 10% of cells synthesizing new DNA. Unfortunately, neither treatment with GSK3β SMI nor GSK3β siRNA rescued the proliferation defects (Figure S6).

### GSK3β inhibition reduces proteolysis and apoptosis in three additional MFS patient lines

Finally, we also sought to determine whether GSK3β inhibition is also beneficial in additional MFS patient iPSC lines. Three additional lines were used for this validation: DE35, DE37, and DE119 (Table S4). These patients were diagnosed with MFS and experienced an aortic event, either dissection/rupture, or had surgery to replace a part of the aorta. These patient lines were reprogrammed into iPSCs, differentiated into VSMCs and treated with GSK3β-targeting SMI AZ3 at 1 μM as was done previously. AZ3 was selected over the other compounds as we noted it had the fewest off-target effects (Figure S5; Table S3).

**Figure 6:**
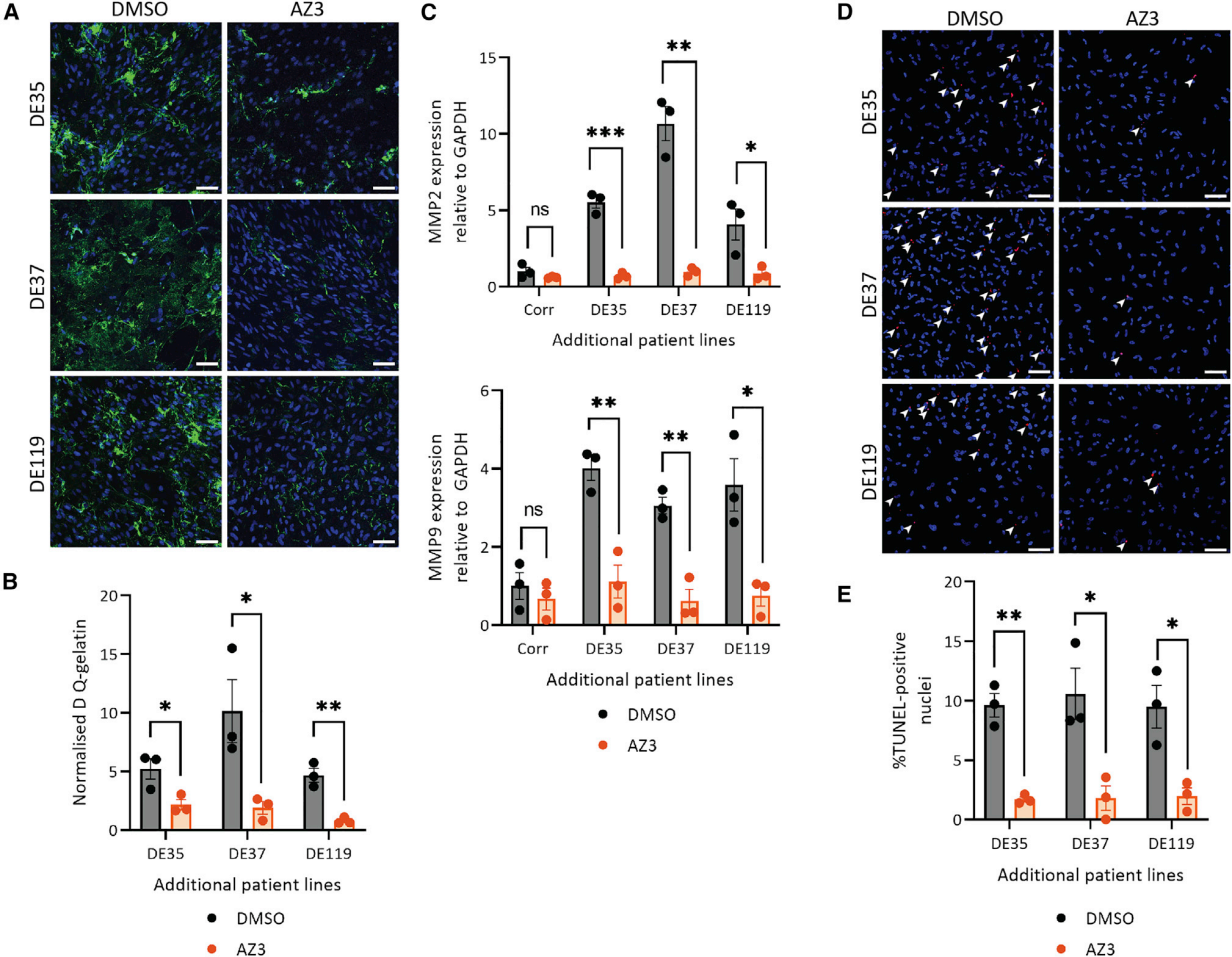
Inhibition of GSK3β using SMI AZ3 in three additional MFS patient lines is beneficial Three additional patient lines—DE35, DE37, and DE119—were differentiated into NC-VSMCs and treated with AZ3. (A–C) Assays were performed as before to assess the effect of GSK3β inhibition on proteolysis as assessed by DQ-gelatin intensity (A and B) and MMP2 and MMP9 mRNA expression (C). (D and E) Apoptosis in these additional lines was also assayed. 150 μm scale bars throughout. n = 3 independent experiments. Data are represented as mean ± SEM.

Cell phenotype was assessed by looking at DQ-gelatin fluorescence and percentage of TUNEL-positive nuclei (Figure 6). The results with these three additional lines support what we have demonstrated with the C1242Y line. In terms of MMP activity, we observed a significant decrease in proteolytic activity after treatment with AZ3 (Figures 6A and 6B). In addition, mRNA expression of MMPs 2 and 9 are also reduced after SMI treatment (Figure 6C), as we had observed previously (Figures 4D and 4E). The apoptotic phenotype of the cells was also reduced after GSK3β inhibition (Figure 6D). Lastly, we wanted to determine whether GSK3β inhibition resulted in any changes in fibrillin-1 deposition. While the MFS patient lines all had abnormal deposition of fibrillin-1, the Corr line displayed uniform and regular fibrils (Figure S7). Upon GSK3β treatment, we observed an improvement in the deposition, although the arrangement of the fibrils is not as regular as in the control. Taken together, this work suggests that GSK3β could be a valuable target to further pursue, showing a beneficial effect in multiple MFS patient lines.

## Discussion

### GSK3β activity in aortic aneurysms

The role of GSK3β in the development of aortic aneurysms is not entirely clear. There is evidence of increased GSK3β phosphorylation in abdominal aortic aneurysms (AAAs) (Krishna et al., 2017). GSK3β was also identified as a likely regulator of pathogenic mechanisms from the analysis of perivascular adipose tissue of patients with AAA (Piacentini et al., 2020). In this study, we demonstrated that GSK3β inhibition was beneficial in our iPSC-derived MFS VSMCs using both multiple SMIs and a genetic approach. In addition, we have validated that SMI inhibition of GSK3β is beneficial in three additional MFS patient lines, suggesting that this may be a common disease mechanism and not a defect specific to the cell line that we used for the initial screening.

GSK3β activity is regulated in an unconventional way compared with most kinases. Many of its targets need to be primed with phosphorylation by another kinase; this post-translational modification will then fit within a groove of GSK3β, allowing it to phosphorylate its target. Inhibitory phosphorylation of GSK3β at the N-terminal Ser9 results in an autologous pseudo-substrate, preventing its binding to primed substrates (Frame et al., 2001). In this study, we have identified that the expression of GSK3β and its phosphorylation at Ser9 is paradoxical, with MFS VSMCs expressing less total GSK3β and having more of the inhibitory Ser9 post-translational modification when compared with the isogenic Corr. Therefore, a big question is why GSK3β inhibition was beneficial despite there being less total GSK3β and more inactivated GSK3β in MFS VSMCs.

There may be explanations that account for this paradox. First, Ser9 phosphorylation may not be a direct readout for GSK3β activity. As reviewed thoroughly by the Jope group, there are four main reasons why this may be: (1) not all GSK3β substrates are primed; (2) GSK3β is often found in complex with other proteins, and *p*-Ser9 does not affect its activity within protein complexes; (3) *p*-Ser9 does not cause total inactivation of its activity; and (4) its subcellular localization could also impact how *p*-Ser9 affects activity (Beurel et al., 2015). In addition, the observed levels of GSK3β could also be the cells’ attempt to incompletely compensate for abnormal cell signaling; treatment with SMIs or siRNA would decrease the need for such compensation, thereby reducing some of the disease phenotypes.

### Downstream targets of GSK3β and clinical perspective

GSK3β is a kinase with numerous interacting partners. While most kinases have an average of 12 interacting partners, GSK3β is predicted to have over 500 targets (Linding et al., 2009). This is due to the unique mechanisms that regulate the activity and availability of GSK3β in a given cell. In this study, we have demonstrated that inhibition of this multi-target kinase with multiple compounds is beneficial, although GSK3β is not a straightforward enzyme to target in a clinical environment. It is ubiquitous and highly expressed in a number of organs, leading to concerns over toxicity and chronic usage. In addition, recent work has emerged on the relevance of a mesenchymal transition of aortic VSMCs in TAAD formation (Chen et al., 2020; Nolasco et al., 2020); GSK3β may have a role in regulating EMT (Zhou et al., 2004), therefore potentially complicating the use of GSK3b inhibitors in treating aortic disease.

Despite these concerns, lithium salts, which include GSK3β as one of their targets (Stambolic et al., 1996), have been used for decades to treat psychiatric disorders (Freland and Beaulieu, 2012), demonstrating the feasibility of long-term GSK3β inhibition at appropriate doses. The therapeutic window of lithium is quite narrow, between 0.4 and 0.8 nmol/L, and doses above this threshold are not well tolerated (Malhi and Berk, 2012). AZD1080, which was also used in our *in vitro* studies under the name “AZ3” (Table 1), has progressed into phase I clinical trials (Georgievska et al., 2013), although it was subsequently abandoned after finding that it resulted in abnormalities in dog gall bladder at that dosage and did not enter phase II trials (Bhat et al., 2018). Currently, one GSK3β inhibitor, Tideglusib, has successfully gone through phase II trials for myotonic dystrophy (Horrigan et al., 2020).

The question of toxicity is particularly important for treating a life-long disease such as MFS. Upon diagnosis with the disease, patients will likely continue drug treatments for the rest of their lives. As such, treatment regimens have to be extremely well tolerated. Losartan was unsuccessful in clinical trials, yet the very similar drug irbesartan retarded the rate of aortic growth compared with the placebo (Mullen et al., 2020). One key difference between losartan and irbesartan is their respective half-lives: the longer half-life of irbesartan increases its bio-availability compared with losartan, suggesting that insufficient dosage could be one of the reasons behind poor performance of losartan in clinical trials.

With this in mind, a GSK3β inhibitor alone may not be appropriate for treating MFS—instead, combining GSK3β inhibitors with other drugs, all at lower concentrations, would allow us to target multiple signaling abnormalities while still being well tolerated by patients. This may be particularly important given that some cellular abnormalities, such as proliferation and fibrillin-1 deposition, are not rescued fully by GSK3β inhibition and may benefit from additional compounds to cover those weaknesses. Alternatively, there are likely a multitude of downstream effectors that are currently unknown but may be more specific in the context of MFS and other aortic diseases. Proteomics and other unbiased approaches could be used to identify downstream effectors of the GSK3β pathway in the aorta, which may be more tractable to therapeutic intervention.

### Further optimizing the phenotypic screen

Despite recent demonstration of the benefits of angiotensin II receptor blockers, there is still enormous scope for additional therapeutic intervention since irbesartan did not reverse or halt progressive aortic dilatation. Moreover, the experience with losartan suggests that mouse models can be difficult to align with the results of human clinical trials. As a result, screening for potential therapeutic compounds solely in mice is not efficient.

Using this human *in vitro* screen, we have identified GSK3β among other interesting targets. The screen in this assay was performed in a medium-throughput manner where cells were cultured in a 24-well format. Scaling cell culture to 96-well plates for higher throughput was unsuccessful for the initial drug screen; we hypothesize that this is an effect of cell density and insufficient signal-to-noise ratio. We have since managed to perform MMP activity assays in a 96-well format by modifying the culture conditions. It should be noted that MMP activity is quite a non-specific readout for MFS. However, we believe that this is an important feature for our drug screen as it is a straightforward assay that can be used to rapidly eliminate large numbers of uninteresting compounds in an initial screen before more specific and complicated assays are used on the short-listed compounds. This strategy could therefore be used for future studies, allowing us to interrogate even larger libraries of compounds, including the full 14,000 compound library from AstraZeneca; obtain an even larger list of potential targets; improve the power of our compound screens; and identify compounds that are more straightforward to transition into human clinical trials.

Our screen has identified a number of compounds that were able to decrease the proteolytic phenotype of MFS. However, even from our relatively modest drug screen of 1,022 compounds, we identified 538 unique putative drug targets where pIC50 values were above 6. In future studies, we aim to interrogate a much larger cohort of SMIs, which in turn will also generate a larger list of potentially drug-able targets. How this large list is managed and how one decides which compounds are worthwhile pursuing will be a challenge. One strategy could be to compare the list of interesting drug targets with lists of SNPs that have been identified in genome-wide studies. Another strategy could be to follow up the compound screen with a genetic screen, utilizing CRISPR for example, to narrow down the list of putative targets even further.

When further optimized, we envisage that this screening strategy could be an extremely valuable toolkit for future studies. As discussed above, a combinatorial approach to treating disease could be explored where patient-derived VSMCs are treated with different combinations of drugs, all at doses chosen to minimize *in vivo* toxicity. Additional patient lines could also be studied to identify signaling pathways that are commonly disrupted—these particular pathways could potentially be very interesting when considering new clinical trials. Finally, it could be expanded toward other aortic diseases that exhibit abnormalities in MMP activity.

## Experimental procedures

### Resource availability

#### Corresponding author

Please contact Sanjay Sinha (ss661@cam.ac.uk).

#### Materials availability

iPSC lines used in this study are available from the lead contact with a completed materials transfer agreement.

#### Data and code availability

The data supporting the results of this study are available within the main paper and supplemental information.

### Cell culture

Isogenic control and patient iPSC lines were derived, cultured, and differentiated as described previously (Cheung et al., 2012; Granata et al., 2017; Serrano et al., 2019). The patient line contains a C1242Y mutation in FBN1, and the original fibroblast line was obtained from Coriell’s cell bank (GM21943), and the isogenic control was generated using CRISPR-Cas9. Additional patient lines (denoted DE35, DE37, and DE119) were obtained from Sonalee Laboratory, St George’s Hospital, London, UK, with the help of Dr. Anne Child. These were received as fibroblasts and were reprogrammed using Sendai Virus v.2.0, as performed previously (Granata et al., 2017) and under research ethics committee approval (11/EE/0053).

Briefly, iPSCs were cultured and maintained on vitronectin-XF (Stem Cell Technologies) and E8 media (DMEM/F12 [Gibco]; Insulin-Transferrin-Selenium Supplement [Gibco]; 0.44 μM L-ascorbic acid [Sigma]; 0.05% sodium bicarbonate [Sigma-Aldrich]; 25 ng/mL FGF2 [R&D Systems]; and 1.74 ng/mL transforming growth factor β [TGF-β; Peprotech]). For differentiation, a chemically defined medium (CDM) (50% IMDM [Gibco]; 50% Ham’s F12 Nutrient Mix [Gibco]; chemically defined lipid concentrate [Life Technologies]; 15 μg/mL transferrin [R&D Systems]; 7 μg/mL insulin [Sigma-Aldrich]; 450 μM monothioglycerol [Sigma-Aldrich]; and 1 mg/mL poly-vinyl alcohol [Sigma-Aldrich]) was supplemented with different cytokine and inhibitors. NC differentiation was initiated by culturing iPSC colonies in FSB media [CDM with 12 ng/mL FGF2 (R&D Systems) and 10 nM SB431542 (R&D Systems)] for 4 days, before being split into single cells and further cultured on 0.1% gelatin-coated plates. These NC were cultured and differentiated into NC-VSMCs in PT media (CDM with 10 ng/mL PDGF-BB [Peprotech] and 2 ng/mL TGF-β [Peprotech]) for 12 days. After differentiation, VSMCs were matured for 2 weeks in DMEM/F12 (Gibco) containing 10% fetal bovine serum (Gibco) before being used in assays (Figure 1A). Although NC-VSMCs are used in this work, we refer to them as “VSMCs” throughout for simplicity.

### SM screen

SMs were obtained from AstraZeneca, and 1,022 drugs were selected out of their library of 14,000 compounds (Parafati et al., 2020). These SMs were diluted from 10 mM stock in DMSO to a final concentration of 1 μM in MEF media. Control and MFS VSMCs were seeded onto 0.1% gelatin-coated 24-well plates. The following day, the 96 h treatment with SMIs began, with a medium refresh halfway through. On day 4, cell culture medium was then collected to assay for MMP activity using the SensoLyte 520 Generic MMP Assay Kit Fluorometric (Anaspec) according to the manufacturer’s instructions for protocol B. Briefly, supernatants were incubated with 1 mM AMPA for 3 h at 37°C, and 50 μL was transferred to a 96-well plate. 50 μL of the included MMP substrate solution was added to each well and further incubated for 1 h at room temperature, after which 50 μL Stop Solution was added to terminate the reaction. Fluorescence intensity, corresponding to MMP activity, was measured at Ex/Em = 490/520 nm on a plate reader.

### siRNA transfection

siRNA knockdown was performed in Opti-MEM media (Gibco) and Dharmafect 1 Transfection Reagent (Horizon Discovery). A non-specific siRNA (ON-TARGETplus; Horizon Discovery) was used as a control alongside siRNA against GSK3-β (Invitrogen). Knockdown in wells of a 6-well plate was performed by incubating 40 nM siRNA with Dharmafect 1 for 20 min before applying to cells. The next day, cell culture media was refreshed, and cells were grown for another 3 days before downstream experiments.

### DQ-gelatin assay

DQ-gelatin fluorescein conjugate (Invitrogen) was dissolved in water to 0.5 mg/mL and used to coat Ibidi 8-well chambered slides or 96-well plates for 24 h at 4°C. Dishes were washed twice with phosphate-buffered saline (PBS) before seeding 15,000 VSMCs. The following day, cells were treated with either SMIs or transfected with siRNA for 96 h before washing with PBS and fixing in 4% PFA (Alfa Aesar) for 10 min at room temperature. Fixed cells were subsequently imaged using a Zeiss LSM 710 confocal microscope. Resulting images were processed and quantified in ImageJ. DQ-gelatin fluorescence intensity was determined after image processing and thresholding. The number of nuclei was also determined after initial processing and analysis of particles. All described image processing and quantification steps were performed using a macro for automated and unbiased analysis.

### Gelatin zymography

VSMCs were seeded in 6-well plates and began treatment with drugs. After 4 days without any media changes, cell supernatants were collected and spun down to remove any debris and floating cells. Supernatant protein content was then quantified using the BCA assay (Pierce) and bovine albumin protein standards. After protein quantification, sample concentrations were normalized prior to mixing with a non-reducing sample buffer. Next, 7.5% SDS-containing polyacrylamide gels with 4 mg/mL porcine skin gelatin (Sigma-Aldrich) were cast using the Bio-Rad system, and 5 μg supernatant was loaded into the wells. Gels were run at 100 V for approximately 2 h before they were incubated in washing buffer (2.5% Triton X-100, 50 mM Tris-HCl [pH 7.5], 5 mM CaCl_2_, 1 μM ZnCl_2_] for 2 × 30 min with gentle agitation at room temperature. Gels were then rinsed in incubation buffer (1% Triton X-100, 50 mM Tris-HCl [pH 7.5], 5 mM CaCl_2_, 1 μM ZnCl_2_) for 10 min, before the incubation buffer was replenished and the gels incubated at 37°C for 24 h with gentle agitation. The gels were then incubated in staining solution (40% methanol, 10% acetic acid, 0.5% w/v Coomassie blue [Sigma-Aldrich]) for 1 h with agitation before being rinsed in ddH_2_O and further incubated with destaining solution (40% methanol, 10% acetic acid) until digested bands became visible. The resulting gel was then scanned, and band intensity was quantified using ImageJ.

### TUNEL staining

VSMCs were seeded onto 0.1% gelatin-coated plates and were treated with either SMIs or transfected with siRNA for 96 h. TUNEL staining to identify apoptotic cells was performed using the *In Situ* Cell Death Detection Kit (Roche) according to the manufacturer’s instructions. Positive controls were obtained by treating cells with 3 U/mL DNase I (Sigma-Aldrich). Tiled images were taken using a Zeiss LSM 710 confocal microscope and quantified in ImageJ using a macro. After image processing, the number of TUNEL-positive nuclei was quantified.

### RNA extraction and qRT-PCR

After washing the cells with PBS, RNA extraction was performed from cells growing in 12-well plates using the GenElute Mammalian Total RNA Miniprep Kit (Sigma-Aldrich) according to the manufacturer’s instructions for extraction from adherent cells. After quantification, reverse transcription was performed using the Maxima First Strand cDNA Synthesis Kit (Thermo Scientific). qRT-PCR was performed using SYBR Green (Applied Biosystems) with 5 ng cDNA per sample. Experiments were performed with technical triplicates, and gene expression was determined based on the expression of housekeeping gene GAPDH using the ΔCT quantification method.

### Drug target analysis

Drug target analysis was done using R (v.4.0.5) and the following packages: ggplot2, pheatmap, dplyr, tidyr, biomaRt, and clusterProfiler (Durinck et al., 2009; Wickham, 2011; Wickham et al., 2019; Wu et al., 2021; Yu et al., 2012).

### Statistics

Statistical significance was determined using an unpaired two-tailed Student’s t test, with p values <0.05 considered to be significant. Significance is shown throughout the manuscript is as follows: ^∗^p < 0.05; ^∗∗^p < 0.01; ^∗∗∗^p < 0.001; ^∗∗∗∗^p < 0.0001.

## Author contributions

H.D. conceived and performed experiments and analysis and wrote the paper. M.M. and R.G.C.M. performed the drug screen with supervision and guidance from A.G. M.H. performed blotting experiments. M.F. and D.M.S. assisted with the design of the screen, provided compounds, and assisted with analysis. A.C. provided patient phenotypes and cell lines from patients with classical Marfan syndrome, and J.A.A.-M. provided fibrillin-1 mutations. S.S. conceived and supervised the project. All authors reviewed the manuscript.

## Supplemental information

Document S1. Figures S1–S7, Tables S4–S6, and supplemental experimental procedures

Table S1. pIC50 values of drug targets from positive hits from the drug screen

Table S2. Frequency of drug targets among effective SMs from drug screen

Table S3. pIC50 values of drug targets of GSK3β-targeting SMIs used for validation

Document S2. Article plus supplemental information

## References

[bib1] Beurel E., Grieco S.F., Jope R.S. (2015). Glycogen synthase kinase-3 (GSK3): regulation, actions, and diseases. Pharmacol. Ther..

[bib2] Bhat R.V., Andersson U., Andersson S., Knerr L., Bauer U., Sundgren-Andersson A.K. (2018). The conundrum of GSK3 inhibitors: is it the dawn of a new beginning?. J. Alzheimers Dis..

[bib3] Cadigan K.M., Waterman M.L. (2012). TCF/LEFs and Wnt signaling in the nucleus. Cold Spring Harb. Perspect. Biol..

[bib4] Chen P.Y., Qin L., Li G., Malagon-Lopez J., Wang Z., Bergaya S., Gujja S., Caulk A.W., Murtada S.I., Zhang X. (2020). Smooth muscle cell reprogramming in aortic aneurysms. Cell Stem Cell.

[bib5] Cheung C., Bernardo A.S., Trotter M.W.B., Pedersen R.A., Sinha S. (2012). Generation of human vascular smooth muscle subtypes provides insight into embryological origing-dependent disease susceptibility. Nat. Biotechnol..

[bib6] Cheung C., Bernardo A.S., Pedersen R.A., Sinha S. (2014). Directed differentiation of embryonic origin-specific vascular smooth muscle subtypes from human pluripotent stem cells. Nat. Protoc..

[bib7] Chung A.W.Y., Au Yeung K., Cortes S.F., Sandor G.G.S., Judge D.P., Dietz H.C., van Breemen C. (2007). Endothelial dysfunction and compromised eNOS/Akt signaling in the thoracic aorta during the progression of Marfan syndrome. Br. J. Pharmacol..

[bib8] Cross D.A.E., Alessi D.R., Cohen P., Andjelkovich M., Hemmings B.A. (1995). Inhibition of glycogen synthase kinase-3 by insulin mediated by protein kinase B. Nature.

[bib9] Cui J.Z., Harris K.C., Raedschelders K., Hollander Z., Potts J.E., De Souza A., Kiess M., McManus B.M., Bernatchez P., Raffin L.A. (2021). Aortic dimensions, biophysical properties, and plasma biomarkers in children and adults with marfan or loeys-dietz syndrome. CJC Open.

[bib10] Dietz H.C., Pyeritz R.E., Puffenberger E.G., Kendzior R.J., Corson G.M., Maslen C.L., Sakai L.Y., Francomano C.A., Cutting G.R. (1992). Marfan phenotype variability in a family segregating a missense mutation in the epidermal growth factor-like motif of the fibrillin gene. J. Clin. Invest..

[bib11] Durinck S., Spellman P.T., Birney E., Huber W. (2009). Mapping identifiers for the integration of genomic datasets with the R/Bioconductor package biomaRt. Nat. Protoc..

[bib12] Flum M., Kleemann M., Schneider H., Weis B., Fischer S., Handrick R., Otte K. (2018). miR-217-5p induces apoptosis by directly targeting PRKCI, BAG3, ITGAV and MAPK1 in colorectal cancer cells. J. Cell Commun. Signal..

[bib13] Frame S., Cohen P., Biondi R.M. (2001). A common phosphate binding site explains the unique substrate specificity of GSK3 and its inactivation by phosphorylation. Mol. Cell.

[bib14] Freland L., Beaulieu J.M. (2012). Inhibition of GSK3 by lithium, from single molecules to signaling networks. Front. Mol. Neurosci..

[bib15] Galatioto J., Caescu C.I., Hansen J., Cook J.R., Miramontes I., Iyengar R., Ramirez F. (2018). Cell type-specific contributions of the angiotensin II type 1a receptor to aorta homeostasis and aneurysmal disease-brief report. Arterioscler. Thromb. Vasc. Biol..

[bib16] Georgievska B., Sandin J., Doherty J., Mörtberg A., Neelissen J., Andersson A., Gruber S., Nilsson Y., Schött P., Arvidsson P.I. (2013). AZD1080, a novel GSK3 inhibitor, rescues synaptic plasticity deficits in rodent brain and exhibits peripheral target engagement in humans. J. Neurochem..

[bib17] Granata A., Serrano F., Bernard W.G., McNamara M., Low L., Sastry P., Sinha S. (2017). An iPSC-derived vascular model of Marfan syndrome identifies key mediators of smooth muscle cell death. Nat. Genet..

[bib18] Groenink M., Den Hartog A.W., Franken R., Radonic T., De Waard V., Timmermans J., Scholte A.J., Van Den Berg M.P., Spijkerboer A.M., Marquering H.A. (2013). Losartan reduces aortic dilatation rate in adults with Marfan syndrome: a randomized controlled trial. Eur. Heart J..

[bib19] Habashi J.P., Judge D.P., Holm T.M., Cohn R.D., Loeys B.L., Cooper T.K., Myers L., Klein E.C., Liu G., Calvi C. (2006). Losartan, an AT1 antagonist, prevents aortic aneurysm in a mouse model of Marfan syndrome. Science.

[bib20] Hansen J., Galatioto J., Caescu C.I., Arnaud P., Calizo R.C., Spronck B., Murtada S.I., Borkar R., Weinberg A., Azeloglu E.U. (2019). Systems pharmacology–based integration of human and mouse data for drug repurposing to treat thoracic aneurysms. JCI Insight.

[bib21] Horrigan J., Gomes T.B., Snape M., Nikolenko N., McMorn A., Evans S., Yaroshinsky A., Della Pasqua O., Oosterholt S., Lochmüller H. (2020). A Phase 2 study of AMO-02 (Tideglusib) in congenital and childhood-onset myotonic dystrophy type 1 (DM1). Pediatr. Neurol..

[bib22] Ikonomidis J.S., Jones J.A., Barbour J.R., Stroud R.E., Clark L.L., Kaplan B.S., Zeeshan A., Bavaria J.E., Gorman J.H., Spinale F.G. (2006). Expression of matrix metalloproteinases and endogenous inhibitors within ascending aortic aneurysms of patients with Marfan syndrome. Circulation.

[bib23] Khera N., Rajput S. (2017). Therapeutic potential of small molecule inhibitors. J. Cell. Biochem..

[bib24] Krishna S.M., Seto S.W., Jose R.J., Li J., Morton S.K., Biros E., Wang Y., Nsengiyumva V., Lindeman J.H.N., Loots G.G. (2017). Wnt signaling pathway inhibitor sclerostin inhibits angiotensin II-induced aortic aneurysm and atherosclerosis. Arterioscler. Thromb. Vasc. Biol..

[bib25] Lacro R.V., Dietz H.C., Sleeper L.A., Yetman A.T., Bradley T.J., Colan S.D., Pearson G.D., Selamet Tierney E.S., Levine J.C., Atz A.M. (2014). Atenolol versus losartan in children and young adults with marfan’s syndrome. N. Engl. J. Med..

[bib26] Linding R., Jensen L.J., Ostheimer G.J., Van M.A.T.M., Jørgensen C., Miron I.M., Diella F., Colwill K., Elder K., Metalnikov P. (2009). Systematic Discovery of in vivo phosphorylation networks rune. Cell.

[bib27] MacFarlane E.G., Parker S.J., Shin J.Y., Kang B.E., Ziegler S.G., Creamer T.J., Bagirzadeh R., Bedja D., Chen Y., Calderon J.F. (2019). Lineage-specific events underlie aortic root aneurysm pathogenesis in Loeys-Dietz syndrome. J. Clin. Invest..

[bib28] Majesky M.W. (2007). Developmental basis of vascular smooth muscle diversity. Arterioscler. Thromb. Vasc. Biol..

[bib29] Malhi G.S., Berk M. (2012). Is the safety of lithium no longer in the balance?. Lancet.

[bib30] Meijer L., Skaltsounis A.L., Magiatis P., Polychronopoulos P., Knockaert M., Leost M., Ryan X.P., Vonica C.A., Brivanlou A., Dajani R. (2003). GSK-3-Selective inhibitors derived from tyrian purple indirubins. Chem. Biol..

[bib31] Milleron O., Arnoult F., Ropers J., Aegerter P., Detaint D., Delorme G., Attias D., Tubach F., Dupuis-Girod S., Plauchu H. (2015). Marfan Sartan: a randomized, double-blind, placebo-controlled trial. Eur. Heart J..

[bib32] Mullen M., Jin X.Y., Child A., Stuart A.G., Dodd M., Aragon-Martin J.A., Gaze D., Kiotsekoglou A., Yuan L., Hu J. (2019). Irbesartan in Marfan syndrome (AIMS): a double-blind, placebo-controlled randomised trial. Lancet.

[bib33] Murray N.R., Fields A.P. (1997). Atypical protein kinase C ι protects human leukemia cells against drug- induced apoptosis. J. Biol. Chem..

[bib34] Nolasco P., Fernandes C.G., Ribeiro-Silva J.C., Oliveira P.V.S., Sacrini M., de Brito I.V., De Bessa T.C., Pereira L.V., Tanaka L.Y., Alencar A. (2020). Impaired vascular smooth muscle cell force-generating capacity and phenotypic deregulation in Marfan Syndrome mice. Biochim. Biophys. Acta Mol. Basis Dis..

[bib35] Oller J., Méndez-Barbero N., Ruiz E.J., Villahoz S., Renard M., Canelas L.I., Briones A.M., Alberca R., Lozano-Vidal N., Hurlé M.A. (2017). Nitric oxide mediates aortic disease in mice deficient in the metalloprotease Adamts1 and in a mouse model of Marfan syndrome. Nat. Med..

[bib36] Parafati M., Bae S.H., Kirby R.J., Fitzek M., Iyer P., Engkvist O., Smith D.M., Malany S. (2020). Pluripotent stem cell-derived hepatocytes phenotypic screening reveals small molecules targeting the cdk2/4-c/ebpα/dgat2 pathway preventing er-stress induced lipid accumulation. Int. J. Mol. Sci..

[bib37] Pepper J., Izgi C., Golesworthy T.J., Takkenberg J.J.M., Treasure T. (2020). Personalised external aortic root support (PEARS) to stabilise an aortic root aneurysm. Br. J. Cardiol..

[bib38] Piacentini L., Chiesa M., Colombo G.I. (2020). Gene regulatory network analysis of perivascular adipose tissue of abdominal aortic aneurysm identifies master regulators of key pathogenetic pathways. Biomedicines.

[bib39] Polychronopoulos P., Magiatis P., Skaltsounis A.L., Myrianthopoulos V., Mikros E., Tarricone A., Musacchio A., Roe S.M., Pearl L., Leost M. (2004). Structural basis for the Synthesis of indirubins as potent and selective inhibitors of glycogen synthase kinase-3 and cyclin-dependent kinases. J. Med. Chem..

[bib40] Serrano F., Bernard W.G., Granata A., Iyer D., Steventon B., Kim M., Vallier L., Gambardella L., Sinha S. (2019). A novel human pluripotent stem cell-derived neural crest model of treacher collins syndrome shows defects in cell death and migration. Stem Cells Dev..

[bib41] Stambolic V., Ruel L., Woodgett J.R. (1996). Lithium inhibits glycogen synthase kinase-3 activity and mimics wingless signalling in intact cells. Curr. Biol..

[bib42] Teixido-Tura G., Forteza A., Rodríguez-Palomares J., González Mirelis J., Gutiérrez L., Sánchez V., Ibáñez B., García-Dorado D., Evangelista A. (2018). Losartan versus atenolol for prevention of aortic dilation in patients with marfan syndrome. J. Am. Coll. Cardiol..

[bib43] Veeman M.T., Slusarski D.C., Kaykas A., Louie S.H., Moon R.T. (2003). Zebrafish prickle, a modulator of noncanonical Wnt/fz signaling, regulates gastrulation movements michael. Curr. Biol..

[bib44] Wagman A.S., Johnson K.W., Bussiere D.E. (2004). Discovery and development of GSK3 inhibitors for the treatment of type 2 diabetes. Curr. Pharm. Des..

[bib45] Wickham H. (2011). The split-apply-combine strategy for data analysis. J. Stat. Soft..

[bib46] Wickham H., Averick M., Bryan J., Chang W., McGowan L., François R., Grolemund G., Hayes A., Henry L., Hester J. (2019). Welcome to the tidyverse. J. Open Source Softw..

[bib47] Wu T., Hu E., Xu S., Chen M., Guo P., Dai Z., Feng T., Zhou L., Tang W., Zhan L. (2021). clusterProfiler 4.0: a universal enrichment tool for interpreting omics data. Innovation.

[bib48] Xie J., Guo Q., Zhu H., Wooten M.W., Mattson M.P. (2000). Protein kinase C iota protects neural cells against apoptosis induced by amyloid β-peptide. Brain Res. Mol. Brain Res..

[bib49] Yu G., Wang L.G., Han Y., He Q.Y. (2012). ClusterProfiler: an R package for comparing biological themes among gene clusters. OMICS.

[bib50] Zhong L., Li Y., Xiong L., Wang W., Wu M., Yuan T., Yang W., Tian C., Miao Z., Wang T. (2021). Small molecules in targeted cancer therapy: advances, challenges, and future perspectives. Signal Transduct. Target. Ther..

[bib51] Zhou B.P., Deng J., Xia W., Xu J., Li Y.M., Gunduz M., Hung M.C. (2004). Dual regulation of Snail by GSK-3β-mediated phosphorylation in control of epithelial-mesenchymal transition. Nat. Cell Biol..

