# Supplementary material for: A phenotypic screen of Marfan syndrome iPSC-derived vascular smooth muscle cells uncovers GSK3β as a new target
Source: Stem Cell Reports. 2023 Jan 19;18(2):555–69. doi: 10.1016/j.stemcr.2022.12.014 (PMC9968988; doi:10.1016/j.stemcr.2022.12.014)
Supplement: Document S1. Figures S1–S7, Tables S4–S6, and supplemental experimental procedures [file mmc1.pdf]

Stem Cell Reports, Volume *18*

Supplemental Information

A phenotypic screen of Marfan syndrome iPSC-derived vascular smooth muscle cells uncovers GSK3β as a new target

Hongorzul Davaapil, Madeline McNamara, Alessandra Granata, Robyn G.C. Macrae, Mei Hirano, Martina Fitzek, J.A. Aragon-Martin, Anne Child, David M. Smith, and Sanjay Sinha

Supplemental Figure 1

A)

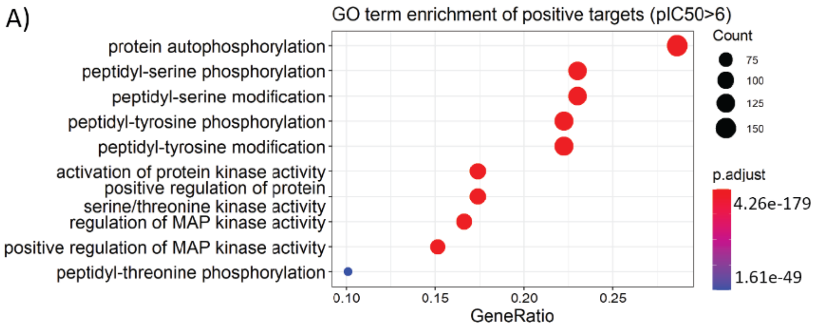

B)

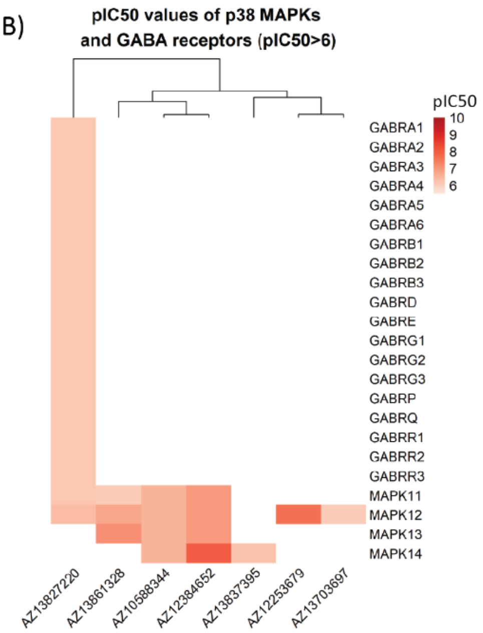

C)

ID	Description	pvalue	p.adjust	qvalue	Count
hsa04010	MAPK signaling pathway	3.50E-39	8.72E-37	2.95E-37	82
hsa04068	FoxO signaling pathway	5.03E-30	6.26E-28	2.12E-28	49
hsa04012	ErbB signaling pathway	2.47E-29	2.05E-27	6.93E-28	40
hsa04722	Neurotrophin signaling pathway	5.77E-29	3.59E-27	1.21E-27	46
hsa05135	Yersinia infection	6.85E-23	3.41E-21	1.15E-21	43
hsa05161	Hepatitis B	2.21E-22	9.17E-21	3.10E-21	46
hsa04660	T cell receptor signaling pathway	5.65E-22	2.01E-20	6.79E-21	37
hsa04360	Axon guidance	6.14E-21	1.91E-19	6.46E-20	47
hsa04510	Focal adhesion	1.28E-20	3.53E-19	1.19E-19	49
hsa05170	HIV1 infection	2.38E-20	5.93E-19	2.01E-19	50

D)

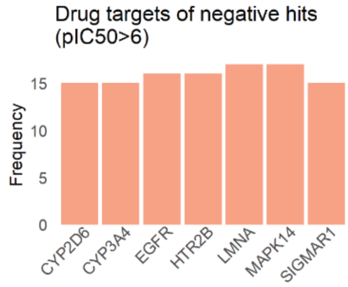

Supplemental Figure 2

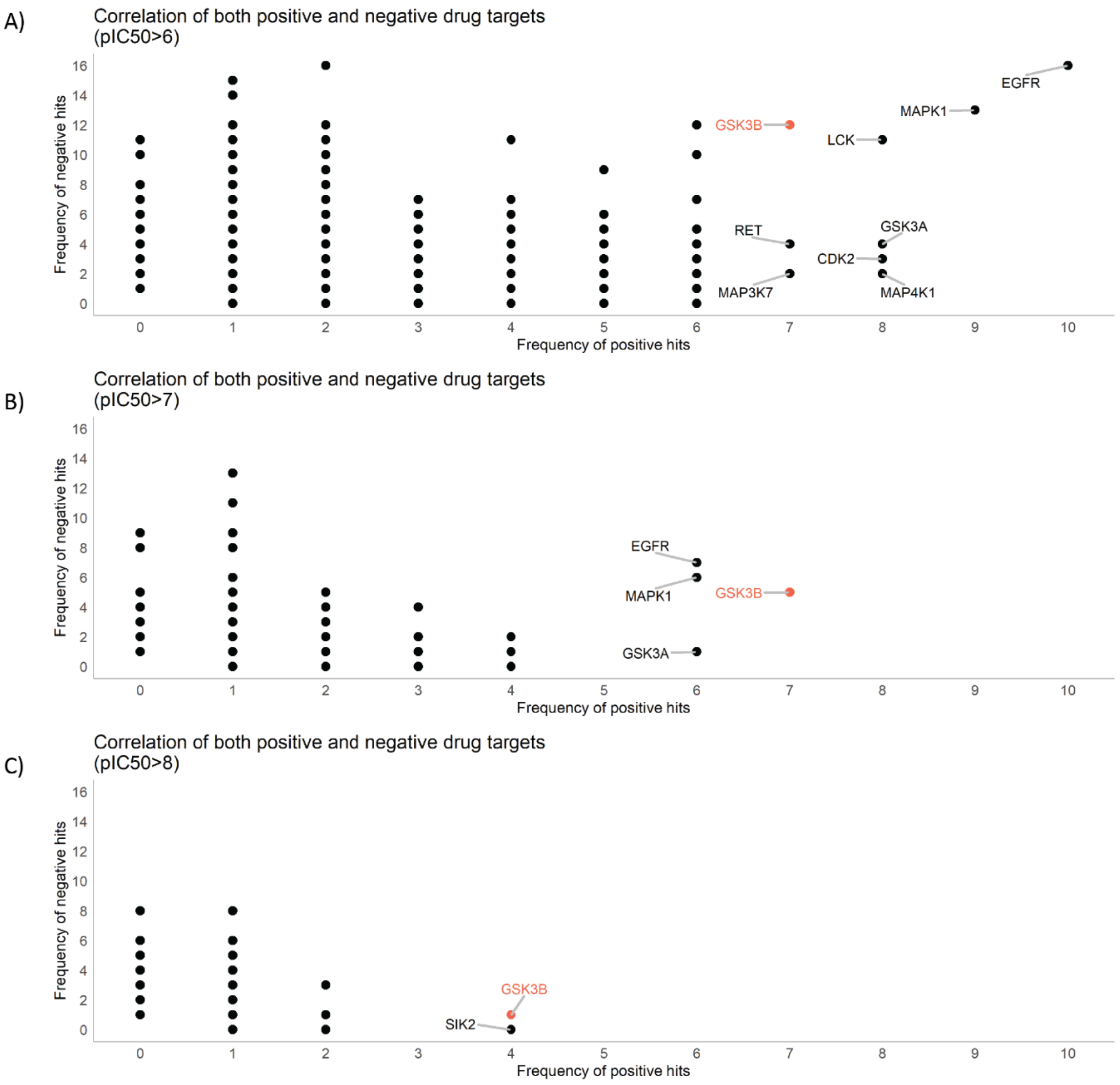

Supplemental Figure 3

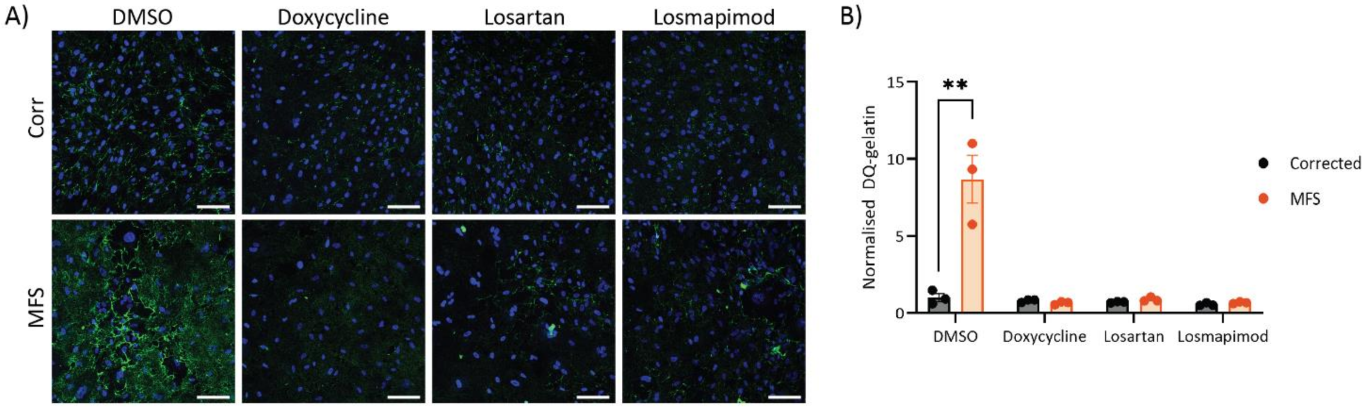

Supplemental Figure 4

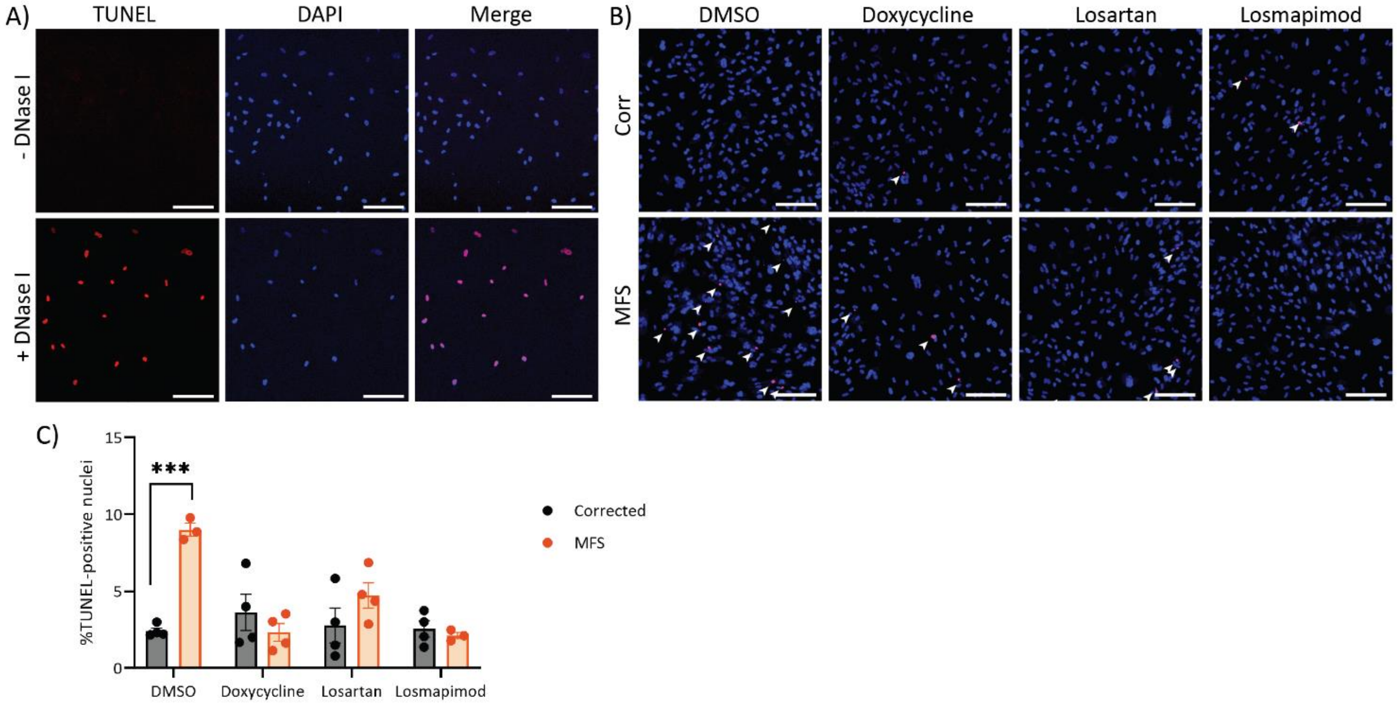

Supplemental Figure 5

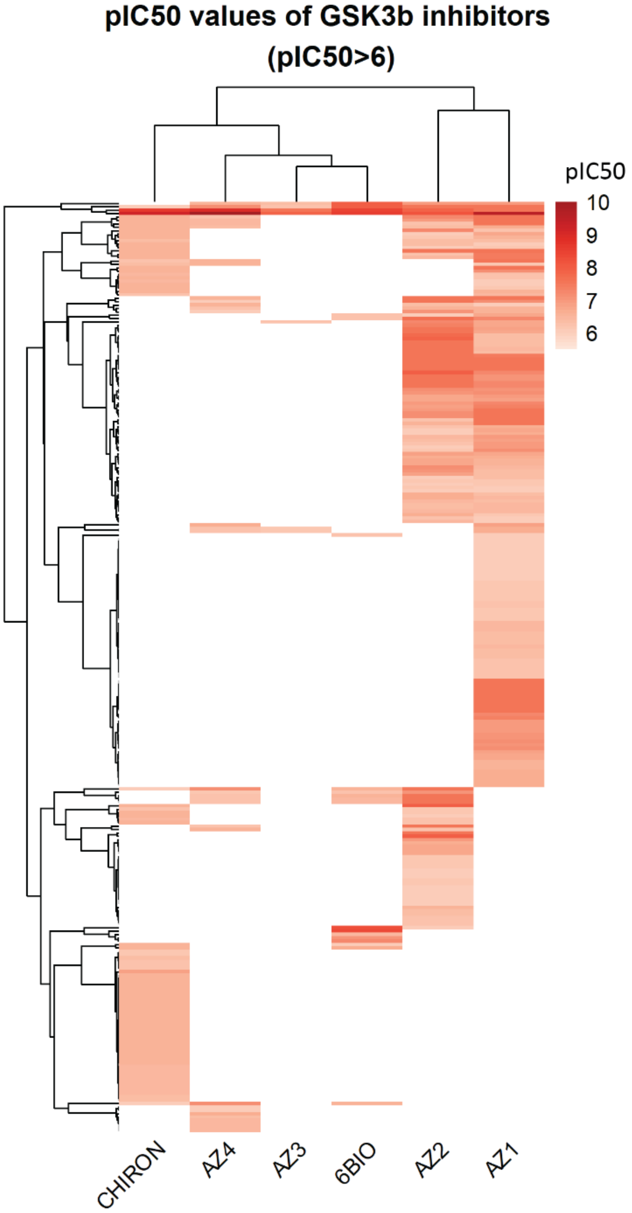

Supplemental Figure 6

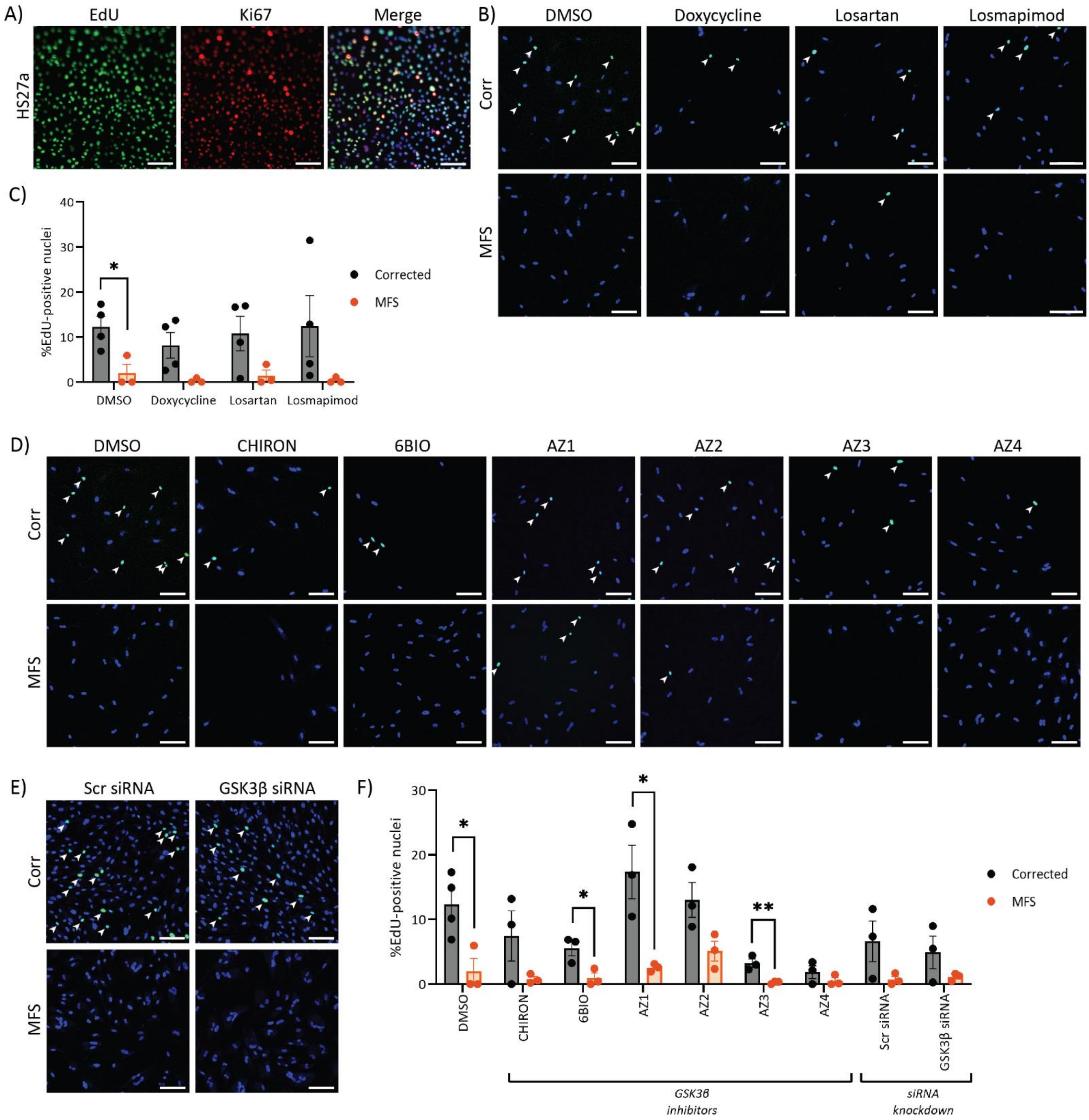

Supplemental Figure 7

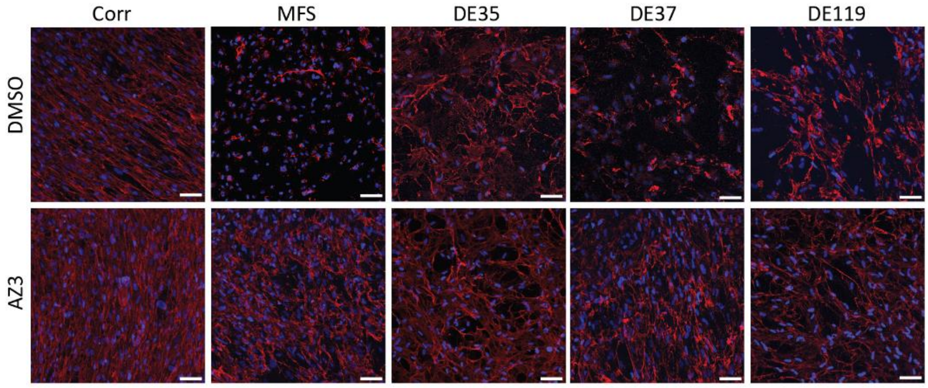

Supplemental Figure legends

Supplemental Figure 1. Computational analysis of positive hits from drug screen. A) GO term enrichment of drug targets indicates that the majority of hits are for kinases. **B)** Previously identified p38 MAPK and GABA receptors were also found to be positive hits from the drug screen. Heatmap illustrates pIC50 values for these interactions. **C)** KEGG Pathway enrichment analysis. **D)** Top recurring targets of the negative hits from the screen does not include GSK3β.

Supplemental Figure 2. Correlation between the number of positive and negative hits filtered by pIC50>6 (A), pIC50>7 (B) and pIC50>8 (C).

Supplemental Figure 3. In-situ zymography with non-GSK3β inhibitors. DQ-gelatin staining **(A)** and quantification **(B)**. 150μm scale bars throughout. n=3. Cells treated under control conditions (DMSO) were also used as controls for Figure 4.

Supplemental Figure 4. TUNEL staining to determine apoptosis. A) Corrected VSMCs treated with DNase I show TUNEL staining. **B)** Treatment with non-GSK3β inhibitors and quantification of TUNEL% nuclei **(C)**. 150μm scale bars throughout. n=3-4. Cells treated under control condition (DMSO) was also used as controls for Figure 5.

Supplemental Figure 5. Off-targets of GSK3β SMI and their pIC50 values. pIC50 values are represented in a heatmap.

Supplemental Figure 6. Proliferation was assessed by incorporation of EdU. A) Highly-proliferative HS27a cells were used as a positive control was used to verify staining conditions. Incorporated EdU was stained in green, Ki67 in red and DAPI in blue. **B)** Effect of non-GSK3β inhibitors on proliferation and **C)** quantification. **D)** Effect of GSK3β inhibitors and siRNA on proliferation and **F)** quantification. n=3-4. 150μm scale bars throughout. Cells treated under control condition (DMSO) were used as controls for panels B and D and their quantifications.

Supplemental Figure 7. Fibrillin-1 deposition is partially restored after treatment for 4 days with GSK3β inhibitors. 150μm scale bars throughout.

Supplemental Tables

Supplemental Table 1. pIC50 values of drug targets from positive hits from the drug screen.

Supplemental Table 2. Frequency of drug targtes amongst effective SMs from drug screen.

Supplemental Table 3. pIC50 values of drug targets of GSK3β-targetting SMIs used for validation.

Cell line	***FBN1* mutation**
DE35	c.1837+5G>C
DE37	Unknown – diagnosis was based on clinical criteria.<br>Reduced fibrillin-1 deposition in patient fibroblasts.
DE119	c.1051C>T; p.(Q351*)

Supplemental Table 4. Additional MFS patient lines used. Where *FBN1* mutations were unavailable, diagnosis was based on clinical criteria for MFS.

Antibody target	Catalogue number	Supplier	Concentration
Calponin-1	C2687	Sigma	1:300
KI67	9129	Cell Signalling Technology	1:300
Fibrillin-1	MAB1919	Millipore	1:100
Goat anti-mouse IgG (Alexa Fluor 594)	A32742	Invitrogen	1:1,000
Goat anti-rabbit IgG (Alexa Fluor 594)	A32740	Invitrogen	1:1,000

Supplemental Table 5. Antibodies used for ICC.

Antibody target	Catalogue number	Supplier	Concentration
GSK3β	9832	Cell Signalling Technology	1:1,000
GSK3β (Ser9)	9336	Cell Signalling Technology	1:1,000
GSK3α	9338	Cell Signalling Technology	1:1,000
β-catenin	9562	Cell Signalling Technology	1:1,000
GAPDH	G9545	Sigma-Aldrich	1:10,000
Mouse secondary	NA931	GE-Healthcare	1:5,000
Rabbit secondary	7074	Cell Signalling Technology	1:2,000

Supplemental Table 6. Antibodies used for western blotting.

Supplemental experimental procedures

Luciferase assay

Cells were seeded onto 12-well plates and transfected with 1µg of M50 Super 8x TOPFlash reporter (Addgene 12456) and 20ng CMV-Renilla Luciferase (Promega) using Lipofectamine 2000 (Invitrogen). The next day, cells were treated with drugs for 4 hours before being harvested and processed using the Dual-Luciferase Reporter Assay Kit (Promega) as per the manufacturer's instructions. After transferring samples to an opaque white 96-well plate, both Firefly and Renilla luminescence signals were measured using a SpectraMax M2e plate reader. M50 Super 8x TOPFlash was a gift from Randall Moon (Addgene plasmid #12456; http://n2t.net/addgene:12456 ; RRID:Addgene_12456).

Immunocytochemistry (ICC)

Cells were washed with PBS and fixed with 4% PFA for 15 minutes. After subsequent washes with PBS, cells were permeabilised with 0.05% triton X-100 before blocking with 10% FBS in PBS. The primary antibody (Supp Table 2) was incubated overnight at 4°C. The next day, samples were washed in PBS and incubated with the secondary antibody (Supp Table 5) for 1 hour at room temperature. Finally, after another wash in PBS, nuclear staining was performed using Hoechst 33342 (Invitrogen) for 10 minutes.

EdU proliferation assay

VSMCs were seeded onto 0.1% gelatin-coated plates and began their 96-hour treatment with either SMIs or transfection with siRNA. They were then incubated with 20µM EdU for 16 hours. EdU staining was performed using the Click-iT™ Plus EdU Cell Proliferation Kit (Invitrogen) as per the manufacturer's instructions. Images were taken using an automated Leica Matrix DMI 6000 microscope. Images were processed and quantified in ImageJ. The number of nuclei stained either for EdU or Hoescht was quantified after initial image processing. All steps were performed using a macro.

Protein extraction and western blotting

Protein was extracted from adherent cells with RIPA buffer (Sigma-Aldrich) supplemented with HALT Protease and Phosphatase Inhibitor (Pierce). After quantification using the BCA Assay (Pierce), lysates were then mixed with 4x Sample Buffer (Bio-Rad) containing β-mercaptoethanol before being denatured at 98°C for 5 minutes. Lysates were subsequently centrifuged at 16,000 x g for 5 minutes, and supernatant corresponding to 4µg protein was then loaded into 4–15% Mini-PROTEAN® TGX™ Precast Protein Gels (Bio-Rad). SDS-PAGE was run for approximately 90 minutes at 80V. Proteins were transferred on a methanol-activated PVDF membrane (Merck) using the wet transfer method running at 60V for 2 hours. Membranes were rinsed with Tris-buffered saline plus 0.1% Tween 20 (TBST) before blocking in 5% BSA for 1 hour before primary antibody incubation was performed overnight at 4°C (Supp Table 6). The following day, membranes were washed 3 times with TBST for 10 minutes each and incubated with HRP-conjugated secondary antibodies (Supp Table 6) for 1 hour at room temperature. Membranes were washed another 3 times with TBST for 10 minutes each before incubating with ECL reagent (Pierce). Films were exposed to membranes before being developed using the Konica Minolta SRX-101A.
